# Obturator Nerve Split for Gracilis Free-flap Double Reinnervation in Facial Paralysis

**DOI:** 10.1097/GOX.0000000000002106

**Published:** 2019-06-19

**Authors:** Alessio Baccarani, Marta Starnoni, Giovanna Zaccaria, Alexandre Anesi, Elisa Benanti, Antonio Spaggiari, Giorgio De Santis

**Affiliations:** From the Division of Plastic Surgery, University of Modena and Reggio Emilia, Modena, Italy.

## Abstract

The use of a double-powered free muscle transfer for facial reanimation has been reported by several authors with different types of nerve coaptation. A new nerve coaptation strategy is presented herein. We performed a 1-stage double-powered free gracilis muscle flap transfer in a patient with long-standing facial paralysis by splitting the obturator nerve and anastomosing the 2 free ends to the contralateral facial nerve (through a sural graft) and to the masseteric nerve. Voluntary movement of the transferred muscle with teeth clenching was observed at 6 months after the operation and a symmetric smile with bilateral elevation of the mouth angle at 10 months. Our limited experience suggests that in case of a large cross-section of the obturator nerve, the latter can be split and sutured to the ipsilateral masseteric nerve and to the contralateral facial nerve with a sural graft by double end-to-end anastomosis.

## INTRODUCTION

Chronic facial paralysis of congenital or iatrogenic origin lasting more than 12 months can be treated by revascularized and reinnervated free muscle flap transfer.

The gracilis free muscle flap, first introduced by Harii et al^1^ in 1976, is often used for several reasons including predictable pedicle anatomy, acceptable donor site morbidity, and favorable muscle microarchitectural features resulting in fast and robust excursion when activated.^2^ This free flap can be innervated by either the contralateral facial nerve, masseteric nerve, or both, in a 1- or 2-stage procedure.

Since its first description,^3^ the use of a double-powered free muscle transfer for facial reanimation has been reported by several authors,^4,5^ with different nerve suture techniques.

A 1-stage double-powered free gracilis muscle flap transfer in a patient with long-standing facial paralysis is described herein by splitting the obturator nerve and anastomosing the 2 free ends to the facial nerve (through the sural graft) and to the masseteric nerve.

## PATIENT

A 53-year-old man presented with facial palsy on the left side in 2016. He had suffered from facial palsy since the age of 20. The etiology was uncertain.

The facial palsy was complete (preoperative House-Brackmann’s score: grade VI) (see video, Supplemental Digital Content 1, which displays preop view of the patient affected by long-standing facial paralysis, http://links.lww.com/PRSGO/B83).

**Video Graphic 1.**
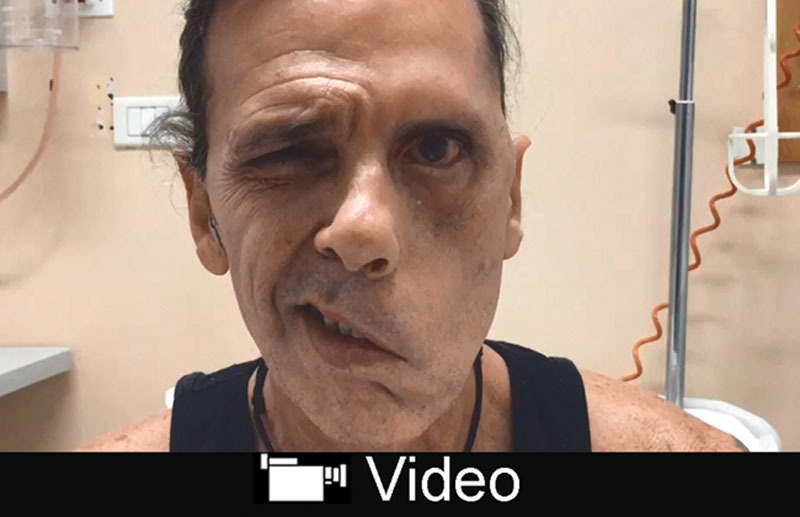
See video, Supplemental Digital Content 1, which displays preoperative view of the patient affected by long-standing facial paralysis, http://links.lww.com/PRSGO/B83.

## SURGICAL PROCEDURE

A myocutaneous facial flap was dissected on the affected side by a subeyelid incision, lengthened to the preauricular region with a 6 cm laterocervical extension.

The facial artery and vein and the superficial temporal artery and vein were exposed. The masseteric motor nerve was identified with gentle dissection through the masseteric muscle, laying 1.5–2 cm deep from the muscle surface.

Next the modiolus was identified medially. Four sutures were thus fixed including modiolus, upper and lower lip, and left philtral column. A subcutaneous pocket was created in the upper lip.

The incision was made on the contralateral unaffected side of the face in a natural line from the tragus to the mandibular angle. The skin and SMAS flap was dissected to identify the facial nerve branches emerging from the parotid anterior border. The functionality of the superior and inferior buccal branches was assessed using an electro-nerve stimulator. A subcutaneous pocket was then created toward the upper lip to receive the cross facial nerve graft. The gracilis muscle flap was harvested. The long-axis length was set to be 1 to 2 cm longer than the subcutaneous pocket in the previously measured recipient site. The widths of the proximal and distal ends were set to be equal to the distance from the mouth angle to the top of the ipsilateral cupid bow on the affected side and the length of the zygomatic arch, respectively.

Based on our measurements, the flap was 3 to 4 cm wide and 8 to 10 cm long.

The distal end of the flap was fixed to the previously set 4 sutures. End-to-end microvascular anastomoses were performed between the flap and facial vessels. The flap was thus revascularized and left in place.

A 26 cm long sural nerve graft was harvested from the leg and then transferred to the face along the previously dissected subcutaneous tunnel. End-to-end epi-perineural suturing was carried out between the obturator and masseteric nerves on the affected side. The cross-section size of the obturator nerve turned out to be twice as large as the masseteric branch at microscopical evaluation after perineural soft tissue debridement. The obturator nerve was therefore split according to perineural soft tissue to respect the fascicles integrity. A double end-to-end epi-perineural anastomosis was then performed connecting the split obturator nerve with the sural graft and the masseteric branch, respectively. A collagen sheet was wrapped around the double anastomosis and fixed with fibrin glue.

The proximal end of the flap was fixed to the zygomatic arch by suturing it to the periosteum and the temporalis fascia. End-to-end epi-perineural suturing was carried out between sural graft and the superior buccal branch of facial nerve on the healthy side. Postoperative course was uneventful, and the patient was discharged on postoperative day 5 after assignment to a facial rehabilitation program.

## RESULTS

At 6 months after the operation, voluntary mouth movement on the paralyzed side was observed with clenching. At 10 months after the operation, a symmetric smile with bilateral elevation of the mouth angles was observed (see video, Supplemental Digital Content 2, which displays postoperative view at 1.5-year follow-up, http://links.lww.com/PRSGO/B84).

**Video Graphic 2.**
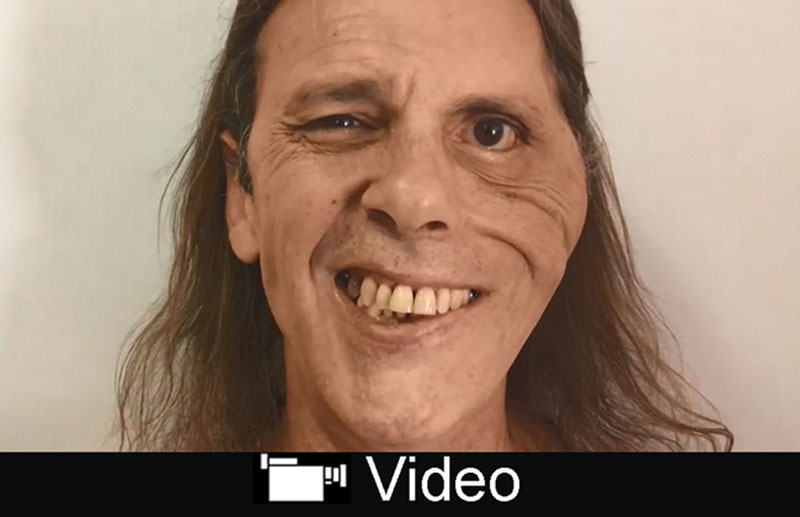
See video, Supplemental Digital Content 2, which displays postoperative view at 1.5-year follow-up, http://links.lww.com/PRSGO/B84.

At 18 months after the operation, an electromyography was performed, revealing that the muscle flap was innervated with the contralateral facial nerve and the ipsilateral masseteric nerves.

## DISCUSSION

In the first described case of free gracilis muscle transfer for treatment of long-standing facial paralysis, the muscle flap was reinnervated by the masseteric nerve.^1^ O’Brien et al^6^ and Vedung et al^7^ reported innervation of the muscle flap with the contralateral facial nerve through the sural nerve graft in 1980 and 1984, respectively.

More recently, 1-stage neurovascular muscle transfer methods involving double innervation were reported.^3–5^ Specifically, Watanabe et al^3^ reported a dual innervation method where the transferred latissimus dorsi flap was innervated with contralateral facial nerve by an end-to-end suture (with the thoracodorsal nerve) and with masseteric nerve via muscular neuratization. Biglioli et al^4^ performed a double innervation by anastomosing the obturator nerve on the ipsilateral masseteric nerve with an end-to-end suture and on the contralateral facial nerve through a sural nerve graft with end-to-side anastomosis. Cardenas-Mejia et al^5^ performed a double innervation suturing the obturator nerve to the contralateral facial nerve through the nerve graft with an end-to-end suture and to the masseteric nerve with an end-to-side suture.

Our approach is a technical modification of the method developed by Biglioli et al.^4^ In essence, the motor nerve is split longitudinally according to the fascicles and the 2 free ends are coapted with end-to-end sutures to the ipsilateral masseteric nerve and to the sural graft. A difficulty with this approach is that the available imaging technologies do not allow to decide since the very beginning if the cross-section size of the gracilis motor nerve is large enough for splitting with respect to the sizes of the others involved nerves. Therefore, the decision can be made only after microscopical inspection during surgery.

As far as physiotherapy is concerned, the treatment was initiated early after patient discharge once the final flap viability was confirmed and continued up to 24 months. Muscle exercises and functional repetitions after relaxing massages were incrementally performed as the muscle started contraction, initially based upon masseteric branch reinnervation. When synkinesis developed, the exercises aimed at controlling the new movement patterns. Through neuromuscular retraining, both facial function and expressions may be substantially improved in the long term. A double-powered muscular reinnervation does not change substantially the physiotherapy protocol; however, the double component has to be kept in mind during training.

**Fig. 1.**
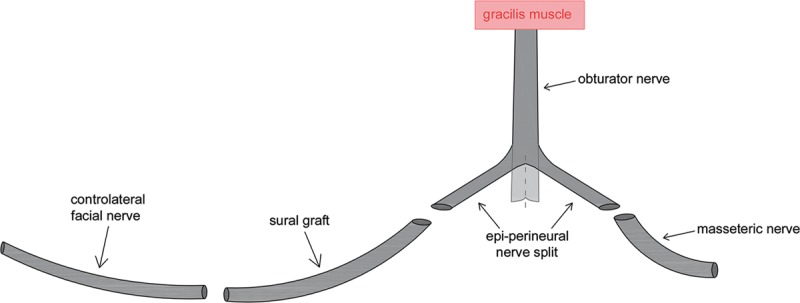
Diagram showing obturator nerve split and double end-to-end anastomosis to the sural graft and omolateral masseteric branch.

**Fig. 2.**
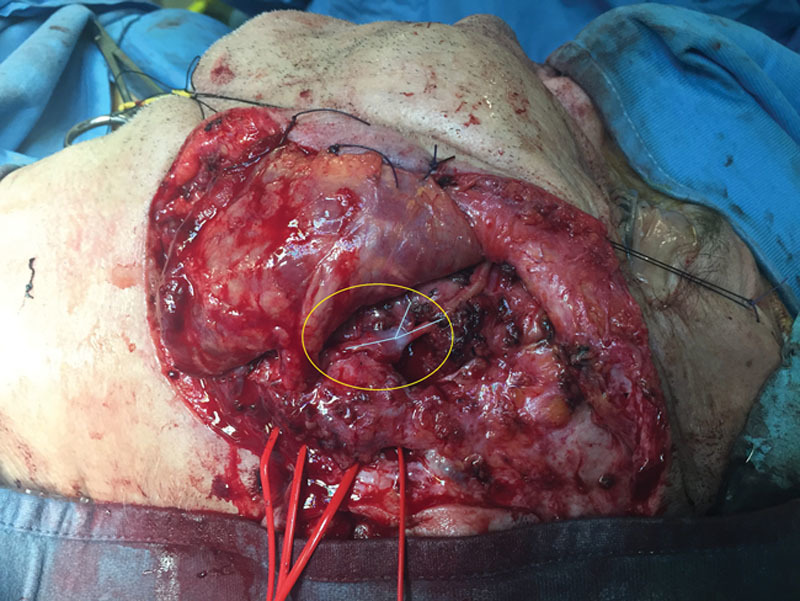
Intraoperatory view after completion of nerve anastomosis.

## CONCLUSIONS

Even if our experience is limited, we think that the longitudinal split of the obturator nerve is advisable when its cross-section is large enough and allows for anastomosing the 2 free ends to the masseteric nerve and to the sural graft. The advantage is that end-to-end anastomoses are technically easier and faster. Moreover, the arrangement of fascicles can be respected preserving a natural microanatomy.
